# Strengthening routine health information systems for analysis and data use: a tipping point

**DOI:** 10.1186/s12913-021-06648-1

**Published:** 2021-09-13

**Authors:** Agbessi Amouzou, Cheikh Faye, Kaspar Wyss, Ties Boerma

**Affiliations:** 1 Institute for International Programs, Department of International Health, Johns Hopkins Bloomberg School of Public Health, MD Baltimore, USA; 2 African Population and Health Research Center, Dakar, Senegal; 3 Swiss Tropical Institute, Basel, Switzerland; 4 University of Basel, Basel, Switzerland; 5 Centre for Global Public Health, Department of Community Health Sciences, Rady Faculty of Health Sciences University of Manitoba, Winnipeg, Manitoba Canada

The global COVID-19 pandemic caused a sudden awakening of the global health community to the crucial importance routine health information systems (RHIS) hold in understanding the effects of the pandemic on health services in low- and middle-income countries (LMICs), offering a tipping point for a significant leap toward stronger systems.

RHIS include the recording and reporting of data on service utilization and provision, morbidity and mortality, and health resources [1]. Although there have been major technological improvements in recent years, the systems remain mostly paper-based at primary health care facility level with health workers combining data compilation functions with service provision duties. Data from facilities are transferred to district health offices, where they are then typically digitized, aggregated, and transferred to the regional and national offices. Data elements are multifold, often fragmented across programs, with multiple registers and forms for recording and compilation. The mandatory monthly reporting, the lack of feedback and systematic data use mean that workers are most concerned about work time use for data compilation and transfer and usually miss the forest for the tree. Due to their continuous and real-time nature and their integration to health service provision, data quality issues in terms of data completeness, timeliness, and accuracy are pervasive.

Public health professionals and many researchers recognize a critical role for RHIS as the backbone of the health system for evidence-based decision-making [2]. First, it generates near real-time data on service delivery inputs, processes and outputs for annual assessment of the performance of health plans and inform management of services. Its value in tracking service utilization trends in crisis situations was shown in West Africa during the Ebola epidemic and is now even more obvious during the COVID-19 pandemic [3]. Second, RHIS data aim to be exhaustive, covering all facilities and ideally also community service provision data, even though private sector reporting is often a challenge. The analysis can be straightforward and standardized, requiring simple comparison of numbers and proportions without a need to account for sampling errors. Third, based on their longitudinal nature and exhaustiveness, some have argued that RHIS represent a good data source for program evaluation based on quasi-experimental design [4]. Similarly, their longitudinal nature and compilation at district level make them fit for effectiveness evaluation of large-scale health programs [5]. Finally, RHIS data are owned and managed by the government, one essential requirement for sustainability and local decision-making.

There is evidence that RHIS have improved in recent years for instance in terms of reporting completeness [6]. There have been substantial improvements in the technology and database organization, in the data content and quality assessment, and in methodologies to analyze these data. A major catalyst has been the transition to the web-based electronic District Health Information System (DHIS-2) by a large number of low- and middle-income countries, mostly in Africa and southern Asia [7–10]. There have been improvements in approaches for data quality assessments, including adjustments for completeness, consistency assessments, identification of outliers and overall data curation [11–13]. Advances are also being made on the methodological front. Examples are alternative ways of estimating denominators for coverage indicators using service statistics, adjustment for reporting completeness, and corrections for missing or outliers [14].

Strengthening RHIS is not a one-off exercise but requires continuous efforts, looping through data collection, data management, quality assessment, analysis and use. It requires building, strengthening and renewing the capacity in countries at all levels. Since 2018, the Countdown to 2030 for Women’s, Children’s and Adolescents’ Health has implemented a series of multi-country health facility data analysis and capacity strengthening workshops, focusing on reproductive, maternal, newborn and child health services, for analysts from country public health research institutions and ministries of health from more than 40 countries in sub-Saharan Africa [6]. The papers in this BMC Health Services Research Supplement build upon this initiative which has further expanded into continued multi-topic country collaboration on evidence generation in reproductive, maternal, newborn and child health and nutrition.

Health facility data rely on data recording and reporting by health workers, duties that take considerable amount of time, including time taken away from direct client and patient contact. Siyam and colleagues provide evidence from five countries in Africa and Asia showing large number of mandatory registers and forms to complete on daily basis, which may take up as much as 30% of health workers’ time [15]. There are too many examples of the introduction of indicators, forms and reports without due consideration of the burden for health workers as well as efficiencies in data collection and reporting.

The majority of papers in this Supplement are concerned with data quality and analysis aspects of RHIS data for coverage estimates at national and subnational levels. A review by Mwinnyaa and colleagues shows the lack of standards for the adjustments of numerators and denominators of coverage estimates and identify priority areas for further work [16]. Four country papers dug into data quality assessment and adjustment issues to assess the potential of developing robust estimates of coverage indicators with routine health facility data.

Maiga and colleagues analyze different options for denominators for coverage statistics and compared the results to household survey results in Sierra Leone [17]. Agiraembabazi and colleagues focus on assessing data quality, adjustments of numerators and denominators and their implications for the coverage of maternal and child health interventions at subnational levels in Uganda, which is one of the countries with the longest use of district estimates for its league tables [18]. Using RHIS data from the Kivu region in DR Congo, Malembaka and colleagues show the usefulness and limitations of facility-based data in generating maternal and child health coverage statistics in fragile settings [19]. Comparing the consistency in immunization coverage estimated from RHIS and survey data in Ethiopia, Pond and colleagues find major discrepancies due to data quality issues [20].

An important advance in the use of facility data for coverage estimates is the use of geospatial analysis to obtain denominator data for maternal and child health indicators [21]. South Africa has strong tradition of using district level health facility data for coverage tracking. The paper by Day and colleagues shows how the RHIS data in South Africa can be used to construct an index to monitor universal health coverage at district level [22].

Tanzania’s routine health information system includes individual level data on sex, age and cause of death in health facilities since 2016. Nyondo and colleagues apply a systematic data quality assessment of the causes of death data reported by health facilities in Tanzania and assess the effects of interventions to improve data quality [23].

Use of facility data for local decision-making is the goal but evidence is extremely sparse. Prakash and colleagues find evidence of an association between data review meetings, data related decision-making and service coverage at district level in India [24].

Several well-understood and strong principles have been laid out by WHO for the development and strengthening RHIS [1]. We emphasize a few additional points in this special issue of BMC health service research. First, the very first principle is the importance of country leadership and ownership and the commitment to transparency and accountability. However, it must be clear where accountability for the performance of the system lies. The uncoordinated clutter of international advisors, partners, researchers, donors and country stakeholders that have their hands in the country RHIS system must be accountable to the system in the same way as the owner. All must strive to support the development and better performance of the system. Second, RHIS must respond to country needs and continue to adapt to changing epidemiology of the country. In this regards the wealth of evidence generated recently by the Every Newborn Action Plan from testing several coverage indicators around the time of birth is a welcome step toward this need and an example to support and emulate [25, 26]. Third, the false dichotomy often entertained between RHIS and population-based data is disingenuous and inefficient. Both are needed, and must be integrated and coordinated to generate data that reinforce each component and support planning processes and the performance assessment of country healthy plans. Low- and middle-income countries have reached a point where stronger and sustained attention must be dedicated to RHIS and major progress is within reach. The COVID-19 pandemic made it a tipping point.

## Data Availability

Not applicable.

## Abbreviations

DHIS: District Health Information System
LMIC: Low- and middle-income countries
RHIS: Routine health information system
WHO: World Health Organization
